# Hereditary Hematopoietic Malignancies: Considerations for Optimizing Diagnosis and Management

**DOI:** 10.1007/s11912-025-01699-7

**Published:** 2025-06-26

**Authors:** Amy M. Trottier, Lea Cunningham, Lucy A. Godley

**Affiliations:** 1 Division of Hematology and Hematologic Malignancy, Department of Medicine, Dalhousie University, Halifax, NS Canada; 2 Immune-Deficiency Cellular Therapy Program, Center for Cancer Research, National Cancer Institute, National Institutes of Health, Bethesda, MD USA; 3 Myeloid Malignancies Program, Center for Cancer Research, National Cancer Institute, National Institutes of Health, Bethesda, MD USA; 4 Robert H. Lurie Comprehensive Cancer Center, Division of Hematology/Oncology, Department of Medicine, Northwestern University, 303 E. Superior St., Office 3-113, Chicago, IL USA

**Keywords:** RUNX1, Familial platelet disorder with associated myeloid malignancy (FPDMM), DDX41, HHM, Telomere biology disorder, Germline testing

## Abstract

**Purpose of Review:**

Hereditary hematopoietic malignancies (HHMs) were once considered extremely rare. As diagnostic testing indications and methods have evolved, deleterious germline variants associated with increased risk of developing hematopoietic malignances are recognized increasingly. The purpose of this review is to summarize recent advances in knowledge, diagnostic, and treatment approaches for several well-known HHM predisposition disorders.

**Recent Findings:**

Patients often lack classic signs and symptoms typically associated with HHMs, may present at any age, and may not have a suggestive family history. Early identification of causative variants allows for timely anticipatory guidance for patients and family members and has important implications for optimizing treatment decisions.

**Summary:**

HHMs are not rare. With expanded genetic testing along with appropriate germline tissue selection and ancillary testing, predisposition variants can be identified early and inform appropriate surveillance and treatment decisions for patients and their families.

## Introduction

Hereditary hematopoietic malignancies (HHMs) have distinct classification systems and are relatively common, affecting 5–10% of *de novo* cases across the lifespan [1–3]. This percentage is likely an underestimate due to frequent restrictive indications for germline testing (e.g., positive personal or family history). However, patients often do not present with classic clinical findings or significant family histories, leading to missed diagnosis or incorrect attribution [4]. In addition, *de novo* germline variants are relatively common for particular disorders, and detailed family history may not be revealing. Standard next generation sequencing (NGS) panels may miss copy number variants and splice site variants and are unable to distinguish between germline and somatic variants [5]. Failing to identify an underlying cancer risk disorder has significant consequences for the index patient as well as their family members, including missed opportunities for cancer surveillance, assessment of additional organs at risk for dysfunction, anticipatory guidance, genetic counseling, and cascade testing. Moreover, providing allogeneic hematopoietic stem cell transplantation (HSCT) to patients with undiagnosed germline cancer risk conditions may lead to excessive chemotherapy toxicity or inadvertent utilization of an affected familial donor. Multidisciplinary stakeholders are increasingly recognizing the benefits of universal testing for all patients with malignancy to guide appropriate patient care [6, 7].

## Case 1

A 7-year-old female was seen in the emergency department due to 2–3 months of upper respiratory infection symptoms and progressive fatigue. Complete blood cell count (CBC) was remarkable for pancytopenia, with total white blood cell count (WBC) 1.2 × 10^9^/L, hemoglobin (Hgb) 6.8 g/dL, and platelets 54 × 10^9^/L. Peripheral blood smear was notable for atypical cells, concerning for circulating blasts. A bone marrow biopsy demonstrated erythroid and megakaryocytic dysplasia and 22% myeloblasts, consistent with acute myeloid leukemia (AML). Karyotype was 46XX, and cerebral spinal fluid analysis was unremarkable. The patient was treated with 7 + 3 induction therapy with cytarabine, daunorubicin, and etoposide, but had 7% persistent blasts after the first cycle, and 4% measurable residual disease (MRD) after second induction with a second course of cytarabine, daunorubicin, and etoposide. A myeloid NGS panel revealed a pathogenic variant in *RUNX1* c.602 G > A, p.Arg201Gln at a variant allele frequency (VAF) of 49.7% from bone marrow and 50% from buccal swab. She underwent an allogeneic HSCT using a 10/10 matched unrelated donor, but unfortunately, her day + 100 bone marrow biopsy was MRD^+^. Her course was further complicated by multiple refractory infectious complications, and she remained refractory to chemotherapy, unable to undergo a second HSCT.

The patient’s birth history was notable for induced vaginal delivery at 39 weeks gestation to a 28-year-old previously nulliparous female with immune thrombocytopenia (ITP; platelets 48 × 10^9^/L) diagnosed during the pregnancy. The patient’s birth weight was 3.05 kg and length 19.5 cm. Physical exam was negative for congenital malformations. CBC was performed at birth due to the mother’s thrombocytopenia, and the neonate’s platelets were (16 × 10^9^/L), confirmed on peripheral smear. The remainder of the CBC, including mean platelet volume, was normal. Cranial ultrasound was negative for signs of bleeding. She remained well appearing, and there were no concerns for a consumptive or sequestration process. Neonatal autoimmune thrombocytopenia seemed the most likely diagnosis, due to maternal ITP, and was expected to be self-limited with supportive care. She was admitted to the neonatal intensive care unit and received a platelet transfusion with increase to 120 × 10^9^/L. Her platelets decreased to 29 × 10^9^/L by day 4, and she received a second platelet transfusion, with increase to 140 × 10^9^/L, and was discharged home. Her platelets remained > 110 × 10^9^/L at her subsequent pediatrician visits through one year of age when she was lost to follow-up. In addition to the mother’s history of ITP, family history was notable for a maternal aunt with a history of ITP, who was also treated successfully for leukemia of unknown type.

### Case 1 Discussion: Familial Platelet Disorder with Associated Myeloid Malignancy

Familial platelet disorder with associated myeloid malignancy (FPDMM) is caused by heterozygous deleterious germline *RUNX1* variants [8]. Deleterious germline *RUNX1* variants were initially thought to affect a few hundred families [9]. However, current estimates place the likely number of such families closer to 5,500 worldwide [10]. Deleterious variants in *ANKRD26* and *ETV6* are less common and responsible for two other familial platelet disorders associated with hematopoietic malignancies (HMs) [1, 2].

**Fig. 1:**
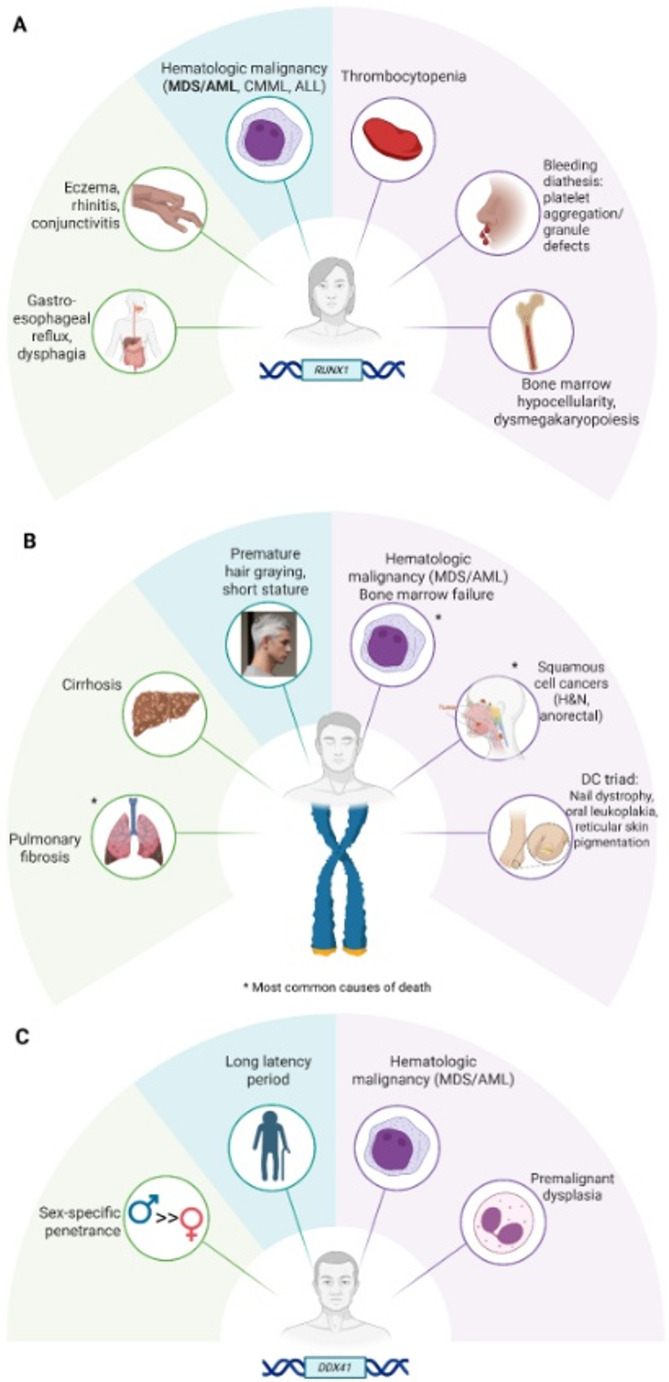
Spectrum of common clinical phenotypes of various HHM syndromes. (**A**) RUNX1 Familial platelet disorder with associated myeloid malignancy phenotype, (**B**) Telomere biology disorder phenotypes, (**C**) Germline DDX41-mediated myeloid malignancies. AML, acute myeloid leukemia; ALL, acute lymphoblastic leukemia; CMML, chronic myelomonocytic leukemia; DC, dyskeratosis congenita; H&N, head and neck; MDS, myelodysplastic syndrome. Created in BioRender. Trottier, A. (2025) https://BioRender.com/k9txru2; https://BioRender.com/9ha9xp0 ; https://BioRender.com/auxwuq9

From a clinical perspective, patients with deleterious germline *RUNX1* variants often present with quantitative platelet defects such as mild to moderate thrombocytopenia although some patients may have normal platelet counts. Patients may also have bleeding and bruising signs and symptoms out of proportion to their thrombocytopenia due to qualitative platelet defects. Qualitative defects classically manifest on platelet aggregation as ‘aspirin-like’ defects of ATP release in response to low dose ADP and collagen. Some patients also have defects of platelet dense and/or alpha granules as determined by platelet electron microscopy [11]. Patients may present for the first time at any point in the life span from congenital thrombocytopenia, pediatric bruising (which may be confused with non-accidental trauma), bleeding out of proportion to surgical challenges (e.g., odontectomy), pregnancy complications, to issues as older adults [12–14]. Additional phenotypic (e.g., gastrointestinal and allergy/immunology symptoms) and genotypic (e.g., early acquisition of secondary somatic mutations) characteristics have been described through the longitudinal RUNX1 Natural History Study (Fig. 1A) [15, 16]. Prior to the molecular diagnosis of a deleterious germline *RUNX1* variant, many patients were given incorrect diagnoses, such as ITP, familial ITP, platelet storage pool disorder, atypical von Willebrand disease, or Glanzmann thrombasthenia, for an average of 8.5 years and median of 6 years prior to accurate determination of their germline condition [15]. Those with initial missed identification of causative germline *RUNX1* variants were diagnosed during subsequent hematology clinic second opinion visits or referrals for HSCT or surgery, when broader molecular testing was performed (Supplemental Fig. 1) [15, 17]. A subset of patients without hematologic signs or symptoms, or those with subclinical signs or symptoms, were found to have deleterious germline *RUNX1* variants for non-hematology/oncology NGS indications, including work-up for developmental delay [15].

Approximately 35–45% of patients with deleterious germline *RUNX1* variants are expected to develop HMs, most commonly myelodysplastic syndrome (MDS) and acute myeloid leukemia (AML), Juvenile Myelomonocytic Leukemia-like, T-cell acute lymphoblastic leukemia (ALL), and occasionally B-cell ALL and other lymphoid malignancies [18–22]. There is not yet a widely accepted mechanistic explanation for the relationship between RUNX1-associated platelet abnormalities and risk of HM [23]. Patients with deleterious germline *RUNX1* variants without hematologic signs and symptoms are still at risk of developing HM and may have delayed diagnosis of their underlying germline variant because bleeding and bruising symptoms did not bring them to earlier medical attention. It is unclear which deleterious germline *RUNX1* variants are most associated with malignancy, although there is some suggestion that dominant negative variants may be more pathogenic than haploinsufficiency. Patients with myeloid malignancy usually have additional pathogenic somatic mutations associated with risk of malignant progression, similar to MDS/AML patients without germline predisposition syndromes, although there are rare cases where no somatic mutations can be identified [15, 16, 24, 25]. Dysregulated inflammatory states are also hypothesized to potentiate the risk of HM [26, 27]. There is also emerging literature evaluating the relationship between microbiota and development of secondary somatic mutations [28]. The risk of developing HM remains relatively high and may be unrelated to phenotype severity. The genome-first UK BioBank cohort study reported deleterious germline *RUNX1* alleles increase the risk of HMs in general (odds ratio 66) and myeloid malignancies (odds ratio 210) in particular (*p* ≤ 0.001) [29]. Close clinical surveillance of pre-malignant patients with germline *RUNX1* variants is essential to identify individuals with emerging malignant transformation at the earliest stages.

How early individuals should be identified as having a deleterious germline *RUNX1* variant is a matter of debate. In the United States, the Advisory Committee on Heritable Disorders in Newborns and Children (hrsa.gov) specify inclusion criteria for screening for 38 core genes, and individual states up to 26 secondary conditions in which early diagnosis and intervention results in improved outcomes. Inborn errors of immunity and hemoglobinopathies are included, but inherited platelet disorders are not included in primary newborn screening lists.

Early molecular diagnosis of pathogenic (P) or likely pathogenic (LP) germline *RUNX1* variants may have significant advantages. Patients would have the correct diagnosis to guide management and avoid unnecessary and ineffectual treatment for incorrect diagnoses (e.g., ITP). Genetic counseling and cascade testing for family members may be provided. Anticipatory guidance may be provided for patients and families so that they may consider the benefits of wearing medical alert jewelry and are aware of the potential for increased risks of bleeding and bruising with contact sports, planned surgical interventions, or childbirth. Baseline knowledge may also be shared with community stake holders, such as teachers and coaches, who might otherwise suspect non-accidental trauma and unnecessarily refer families to child protective services. Patients and families may also make informed decisions about routine/surveillance hematology visits to monitor for malignant transformation.

The frequency with which individuals are recommended to have surveillance clinic visits is currently determined on a case-by-case basis, although most recommend obtaining a baseline bone marrow evaluation as soon as safely feasible (i.e., considering risks versus benefits of general anesthesia for very young children and/or coupling bone marrow evaluation with any other needed sedated procedures). There are likely benefits of more frequent testing for patients with CBC changes over time, especially with either non-platelet lineages affected or changes in baseline platelet counts. Patients with dominant negative *RUNX1* variants, alleles that change known RUNX1 ‘hot spots’ (e.g., p.Arg201), or those with acquired secondary *RUNX1* somatic mutations may be at higher risk for malignant transformation. Additional secondary somatic mutations linked to PRC1.1 and PRC2 complexes may also be associated with a higher risk of malignant transformation [16, 30]. Early referral for HSCT allows patients to receive transplantation sooner, potentially with better disease control and improved outcomes. Conversely, over-medicalization may be associated with a lower quality of life and increased financial toxicity. Although the gold standard for clinical surveillance remains a bone marrow biopsy, they are invasive and uncomfortable. Development of non-invasive biomarkers with high sensitivity and specificity for impending malignant transformation are needed.

The patient described in Case 1 may have benefited from recognition of her causative germline *RUNX1* variant at birth to help guide treatment and management. Although genetic variants associated with inherited platelet disorders are not currently included in primary newborn screening, these disorders should be strongly considered for secondary newborn screening in patients with congenital signs and symptoms of hematologic dysfunction, even with plausible explanation of neonatal autoimmune thrombocytopenia, especially with positive family history, and persistent quantitative or qualitative platelet defects. This knowledge may have prompted the family to seek specialty care sooner and a hematologist may have recognized the months-long prodrome sometime seen with germline *RUNX1-*related myeloid malignancies. Early recognition may have identified the development of her HM sooner. Refractoriness to standard 7 + 3 induction chemotherapy in the context of a germline variant may have also prompted the medical team to attempt a different course of chemotherapy for re-induction, such as combination of a hypomethylating agent and venetoclax. Better early disease control may have led to earlier and more successful HSCT, and other family members may have also benefited from earlier diagnosis and targeted management.

## Case 2

A 45-year-old male was seen in consultation in the hematology clinic for a mild macrocytic anemia (Hgb 11.0 g/dL, MCV 103 fL) and moderate thrombocytopenia (platelets 75 × 10^9^/L). Past medical history was significant for a recent diagnosis of idiopathic pulmonary fibrosis (IPF). The family history was notable for a sister who passed away from a metastatic oral squamous cell carcinoma at age 32, and a maternal uncle who passed away with IPF. Bone marrow biopsy and aspiration were consistent with a diagnosis of MDS with low blasts, and a Tier 1 variant in *U2AF1* was detected on a myeloid NGS panel. Telomere length (TL) testing by multicolor flow cytometry fluorescence in situ hybridization (flow-FISH) identified TLs between the 1st and 10th percentile for age in lymphocytes. Germline genetic testing was performed from cultured skin fibroblasts revealing a likely pathogenic variant in *TERC*, consistent with a telomere biology disorder (TBD).

### Case 2 Discussion: Telomere Biology Disorders (TBDs)

Various terms have been used to describe telomere disorders, such as short telomere syndrome/disorder, telomeropathy, and TBD. Herein, we will be using the broader term, TBD, as it applies to disorders caused by deleterious germline variants in genes involved in telomere structure, function, and maintenance, resulting in aberrant telomere biology and increased cancer risk as well as a variety of possible clinical manifestations *with or without* telomere lengths being significantly shortened.

Telomeres consist of repeating nucleotide segments (TTAGGG)_n_ at the ends of chromosomes that maintain chromosomal stability [31]. Due to lagging strand DNA replication, nucleotide repeats are lost with each cell division, resulting in sequentially shorter telomeres. When TLs become critically short, cells undergo apoptosis or become senescent. The telomerase complex, an RNA-dependent DNA polymerase that elongates telomeres, in combination with the shelterin complex, CST complex, and additional proteins encoded by genes such as *PARN* and *RTEL1*, aid in maintaining telomeres to prevent premature TL shortening [32, 33]. To date, deleterious germline variants in over a dozen genes have been associated with TBDs with a variety of inheritance patterns (Table 1) [32, 34–37].

**Table 1:** Inheritance patterns of genes associated with telomere biology disorders

Gene Name	Inheritance
*ACD*	AD/AR
*CTC1*	AR
*DKC1*	XLR
*NAF1*	AD
*NHP2*	AD/AR
*NOP10*	AD/AR
*PARN*	AD/AR
*POT1*	AD*/AR
*RPA1*	AD
*RTEL1*	AD/AR
*STN1*	AR
*TERC*	AD
*TERT*	AD/AR
*TINF2*	AD
*WRAP53*	AR
*ZCCHC8*	AD

*Heterozygote deleterious *POT1* variants are associated with cancer predisposition (melanoma and chronic lymphocytic leukemia). AD, autosomal dominant; AR, autosomal recessive; XLR, X-linked recessive

TBDs can present across the age spectrum and with a wide array of clinical manifestations (Fig. 1B). Physical findings can include oral leukoplakia, nail dystrophy, or reticular skin pigmentation, collectively referred to as the dyskeratosis congenita (DC) triad; short stature; premature hair graying; dental carries; and/or periodontal disease [34]. People with a TBD are at increased risk for pulmonary fibrosis, chronic liver disease, bone marrow failure (BMF)/aplastic anemia, MDS/AML, as well as solid tumors, such as squamous cell carcinomas of the head and neck or anogenital region [32–34, 38]. On one end of the spectrum, some TBD patients can present very early in life with developmental delay and congenital anomalies, such as microcephaly and cerebellar hypoplasia as in Hoyerall Hreidarsson syndrome, bilateral exudative retinopathy and intracranial calcifications as in Revesz syndrome, or cerebroretinal microangiopathy with calcifications and cysts as in Coats plus syndrome; whereas some TBD patients have minimal or singular manifestations, such as BMF or pulmonary fibrosis that develops later in life [39]. The classic DC triad is present only in a minority of TBD cases, more often in patients with autosomal recessive TBDs that present earlier in life with a more severe clinical phenotype [32]. Heterozygous carriers of P/LP variants in *TERT*, the most frequently found genetic aberration in TBDs, frequently lack the DC triad [40, 41]. As a result of the broad age spectrum at presentation and varied clinical manifestations, diagnoses can be missed unless appropriate ancillary testing including TL testing and germline genetic testing are performed.

### Challenges with Testing for TBDs

In a patient with suspicious clinical features, the presence of abnormally shortened lymphocyte telomeres (< 1st percentile for age) and/or the finding of a P/LP variant in a gene associated with TBD is diagnostic. However, not all patients with TBDs have very short TLs, and TLs themselves can be influenced by additional factors, making interpretation challenging. In addition to the physiological shortening of telomeres with age, TBDs lead to shorter baseline TLs and accelerated attrition; regenerative replicative stress, such as increased mitotic activity present in proliferative states, BMF, and bone marrow recovery following chemotherapy or hematopoietic stem cell transplant can impact TLs; and DNA damage from ionizing radiation, toxins, smoking, obesity, and inflammation have all been found to result in telomere shortening [42–46]. This presents a particular challenge when trying to interpret TLs in the context of suspected TBD in patients with HM.

TL shortening is accelerated in granulocytes in patients with myeloproliferative neoplasms, however this was not the case for lymphocyte TLs, suggesting lineage specific effects and greater specificity for lymphocytes TLs for TBDs in the context of myeloid diseases [42]. In patients with chronic lymphocytic leukemia (CLL), lymphocyte TLs can vary significantly from abnormally long, as a consequence of increased telomerase activity and slower proliferation in lower risk CLL, to markedly shortened in higher risk, more proliferative, cases [45]. TLs can vary appreciably among patients with MDS, but are in general shorter compared to healthy controls [43, 44]. These MDS studies, however, did not use flow-FISH and were not cell lineage specific, therefore conclusions regarding the impact of MDS on lymphocyte TLs specifically cannot be drawn.

The current standard for clinical TL measurement is flow-FISH, for which a 2-panel assay (total lymphocytes and total granulocytes) or a 6-panel assay (2-panel plus lymphocyte subsets, including naïve T cells, memory T cells, B cells, and NK cells) can be ordered. There are no clear guidelines as to which panel should be ordered, or how to interpret these subsets. Lymphocyte TLs are notably shorter in TBD patients presenting with the classic DC triad compared to non-TBD BMF patients with a sensitivity of over 90% for very low lymphocyte TLs, defined as less than the 1st percentile for age [47]. However, the TLs of memory T cells, NK cells, and granulocytes, in particular, are less sensitive. Very low lymphocyte TL can also help distinguish acquired from TBD-associated aplastic anemia and differentiating TBD patients from normal controls and unaffected relatives with a sensitivity and specificity of 97% and 91%, respectively [48, 49]. These test characteristics were slightly improved to a sensitivity and specificity of 98% and 94%, respectively if three or more of the four lymphocytes subsets were very short [48]. In a population of people with young-onset TBD and BMF, TL testing of lymphocytes alone was highly sensitive and specific with no difference compared to using lymphocyte subsets [50].

Most of these prior studies, however, only included young patients and/or those with more severe phenotypes. Subsequent studies have found that TLs vary with age at presentation as well as with the genotype/phenotype [38, 49]. Although 93% of TBD patients identified before age 40 years had very low lymphocyte TLs, the same was true for only 16% diagnosed after 40, with the majority falling between the 1st and 10th percentile and others within the normal range [49]. TBD patients with pulmonary fibrosis also tend to present later and frequently have TLs above the 1st and even 10th percentile [38]. Patients with heterozygous *PARN* variants and pulmonary fibrosis often have TLs above the 10th percentile [38]. In these cases, the 6-panel assay including lymphocyte subsets may be more informative than total lymphocytes in isolation. As a result, clinicians cannot rely on TLs alone. Rather, a combination of TLs and germline genetic testing is essential for TBD diagnosis, especially for older patients, seemingly unaffected relatives, and those with milder clinical phenotypes (Supplemental Fig. 2).

### Special Considerations for Management and Surveillance

No specific treatment exists for TBDs, and treatment recommendations must be tailored to the individual patient and their associated disease manifestations. For patients with BMF, MDS, or AML, the only curative option is an HSCT. Similarly, for patients with pulmonary fibrosis or cirrhosis, organ transplantation is the only curative option. However, solid organ transplantation and HSCT are associated with increased morbidity and mortality, especially in the setting of other pre-existing organ dysfunction, compared to non-TBD patients, and does not ameliorate other organ impairment [51–53]. If transplant is not an option, antifibrotic agents have been used safely and may slow pulmonary function decline [54]. Androgens have been shown to improve cytopenias in TBD patients with BMF and may increase TLs, although the clinical significance of the latter is unclear and are an option for patients who are not transplant candidates [55, 56].

Due to the heterogeneity in clinical manifestations and the widely variable age at which these manifestations can occur, baseline investigations and regular screening and follow-up as per established guidelines are recommended for all TBD patients as well as asymptomatic individuals or relatives, if they have a confirmed P/LP variant in a TBD-associated gene with an appropriate mode of inheritance [34, 57]. These include, but are not limited to, bone marrow biopsy and aspirate with molecular and cytogenetic testing, pulmonary function testing, liver ultrasound, bone density scan, CBC, and liver enzyme and function tests at baseline. Vaccination against HPV is recommended to reduce risk of HPV-associated squamous cell carcinomas and cervical cancer. Regular cancer surveillance, including annual head and neck cancer screening by an otolaryngologist, annual gynecological examinations, and cervical cancer screening (starting at age 18 or onset of sexual activity, whichever occurs earlier), dental hygiene visits and oral cancer screening every 6 months, CBC every 3–6 months depending on presence or absence of any abnormalities, and bone marrow biopsies with any new or worsened changes on the CBC. Pulmonary function tests are recommended to be performed annually with high resolution CT chest as clinically indicated [53, 57].

It is important to offer family members of a confirmed TBD disorder confirmatory testing so they can initiate the appropriate screening recommendations outlined above. Whereas this screening is not required for heterozygous carriers of variants in genes which are only associated with TBDs in an autosomal recessive manner (e.g.,* CTC1*, *WRAP53*, and *STN1*), it is recommended for carriers of variants in genes which have both autosomal dominant and recessive modes of inheritance (e.g.,* ACD*, *RTEL1*, *TERT*, and *PARN*). A commonly neglected group includes asymptomatic heterozygous parents of a child with severe manifestations found to have compound heterozygous variants or a homozygous variant in a gene with both dominant and recessive TBD associations. The lack of current symptoms and older age relative to the child does not preclude risk of developing potentially severe TBD-associated manifestations or malignancies, as monoallelic variant carriers often develop signs or symptoms later than biallelic patients and earlier age at onset with subsequent generations due to genetic anticipation is a common phenomenon in TBDs.

## Case 3

A 52-year-old man presented to his primary care doctor for his annual physical exam. When the doctor asked the patient about anxiety, the patient confessed that he was paralyzed by worry about his health. He had watched his father, two paternal uncles, and his paternal grandmother develop bone marrow cancers in their 60s, and he was petrified that he would be diagnosed in the coming decade. He was also consumed by guilt that he had already passed on whatever was causing these cancers in his paternal family to his two children. His primary care doctor had just gone to Medical Grand Rounds the week before where the speaker talked about inherited risk to blood/bone marrow cancers, which the primary care doctor had always thought only applied to solid tumors. The primary doctor explained to the patient that this clustering of cancers could be inherited, and he placed a referral to cancer genetics. When the patient was seen by the genetic counselors, they explained that germline cancer risk testing would be best performed on someone who had had cancer, and the patient facilitated a visit for his father. The father had undergone an HSCT from an unrelated donor five years before for MDS, so a skin biopsy was performed to allow growth of skin fibroblasts to obtain DNA reflective of the patient, not the allogeneic donor. DNA was generated from cultured skin fibroblasts and sent for comprehensive cancer risk testing for hereditary cancer risk that included genes in which deleterious variants confer risk for HHMs. The result was available several weeks later and demonstrated a start loss variant in *DDX41*: c.3G > A, p.?. The genetic counselor informed the father, and cascade testing was performed for the family. The initial patient who had prompted the work-up tested negative to his great relief, but his sister was found to share the allele. She asked what she could do to prevent developing a bone marrow-derived cancer like so many of her relatives. She was given the recommendation to undergo a baseline bone marrow examination with cytogenetic/molecular studies, with follow-up several times a year.

### Case 3 Discussion: Germline *DDX41-* Mediated Myeloid Malignancies

There are several unique aspects to the pathophysiology of germline *DDX41-*mediated myeloid malignancies [58–60]. First, they defy the typical assumption that germline cancer risk alleles drive cancer in young individuals. The average age of myeloid malignancies in these people is in the late 60s, the age at which *de novo* MDS/AML occur, although dysplasia is often seen within the bone marrow decades before HMs develop [58]. For this reason, it can be challenging for some health care providers to consider these diseases as possibly germline driven. Second, men with *DDX41* cancer risk alleles are more likely to develop myeloid malignancies 3:1 over women (Fig. 1C). The biological reason for this sex difference is not known. Third, development of myeloid malignancies is accompanied by acquired loss of the wild-type *DDX41* allele, either through loss of chromosome 5q, where the gene is located, or through somatic mutation [58–60]. Several hot-spot acquired mutations have been identified, most commonly the R525H allele [58–60]. Thus, identification of loss of 5q or R525H (or other recurrent acquired mutation) signals molecular progression and should prompt consideration of an allogeneic HSCT. Fourth, *DDX41* cancer risk alleles are common across populations. For example, the start loss variant described above and the Asp140fs allele are more common in people with ancestry from northern Europe, whereas the Ala500fs allele is more common in those from Japan/Korea [59]. Almost all (98%) of truncating *DDX41* alleles are germline. The high frequency of deleterious germline *DDX41* variants in many populations also has implications for the allogeneic HSC donor pool, as there are now many examples of introduction of these alleles at the time of HSCT using an unrelated donor [61]. Because of this, some are advocating now for universal cancer risk testing for all HSC donors regardless of relation to the patient. Fifth, people with deleterious germline *DDX41* variants are at risk for severe graft versus host disease after HSCT, even when donors are known to be *DDX41*^*WT*^, unless post-transplant cyclophosphamide (or likely other strong anti-T cell therapies) are used [62]. Lastly, recent data indicate that in families with both HMs and solid tumors with germline *DDX41* cancer risk alleles, about one-third of them have second germline deleterious variants in other cancer-predisposition genes, which likely drive solid tumor development but may be influenced by the *DDX41* allele [58]. Future studies will hopefully lead to an understanding of the late onset of HM development along with preventive strategies for delaying or even preventing malignancy development.

### Germline Genetic Testing Considerations

Identification of deleterious germline variants and accurate diagnoses of HHM conditions rely on the appropriate acquisition of tissue for germline DNA and selection of a comprehensive genetic testing panel, including all known and relevant HM predisposition genes, with single nucleotide variant and copy number variant (CNV) detection being required (Supplemental Figs. 1 and 2). Panel-based NGS performed on whole-exome, and more recently, whole-genome sequencing (WGS) backbones are becoming less expensive and more commonplace in both commercial and academic genetic testing facilities. Advantages of WGS include greater ease of CNV detection and capability to detect important non-coding regions that may otherwise be missed if using standard WES. In some cases, however, RNA-seq may be required to identify cryptic splice site variants. Long-read sequencing and optical genome mapping are other technologies that are used increasingly, particularly in the setting of clinical trials and research laboratories, but are not yet routine in the clinical setting.

In patients with myeloid malignancy or BMF, for which somatic variants and clonal hematopoiesis are common, the gold standard source of germline DNA is derived from cultured skin fibroblasts [63]. However, requirement for patient travel to clinic, invasive nature of a skin biopsy, necessity of appropriate culturing facilities, lack of medical insurance approval, increased turn-around times due to culturing time, and an increased risk of culture failure in patients with TBDs, all present numerous challenges [64]. Several approaches can be used to mitigate some of these challenges: (i) performing the skin biopsy at the same time and site as a bone marrow biopsy; and (ii) performing two skin biopsies for people with BMF and/or suspected of having a TBD and using one for direct isolation of DNA after extensive washing provides a back-up DNA source in case of culture failure. Recently, there is increased interest in alternative, less invasive, sources of germline DNA in patients with HMs (Table 2). Among these, DNA from hair follicles and nail clippings show the most promise as non-invasive options, but the latter may be contaminated with monocyte DNA and customized extraction protocols are required to ensure sufficient DNA quantity for downstream testing [65–69].

**Table 2:** Summary table of DNA sources for genetic testing in patients with HM

Sample Type	Pros	Cons	Suitable for diagnostic germline testing?
Cultured skin fibroblasts	Source of high quality and quantity DNA. Culturing eliminates contamination by hematopoietic cell derived DNA.	Invasive procedure. Culturing adds additional ~ 4 weeks to turn-around time. May not be available/possible at all centers. Risk of culture failure, especially if delays in culture initiation.	Yes (current gold standard)
Direct skin biopsy (no culturing)	Source of high quality and quantity DNA.	Invasive procedure. Risk of contamination by hematopoietic cell derived DNA	No
Saliva	Non-invasive, accessible, and patients can self-collect.	Contamination by hematopoietic cell derived DNA[68,69]	No
Buccal swab	Non-invasive, accessible, and patients can self-collect.	Contamination by hematopoietic cell derived DNA[68,69]	No
Hair follicles	Non-invasive, accessible, and patients can self-collect. Very low risk for hematopoietic cell contamination	Low DNA quantities. May not be available/possible at all centers.	Yes
Nail clippings	Non-invasive, accessible, and patients can self-collect.	Customized extraction methods required to optimize DNA quantity. May not be available/possible at all centers.	Yes*
Sorted T-cells	Easily accessible	May not be available/possible at all centers. Presence of early somatic variants and/or clonal hematopoiesis variants.	No^†^
Peripheral blood or bone marrow in remission	Easily accessible and minimally invasive	Persistence of somatic variants despite morphologic remission. Presence of clonal hematopoiesis variants. May delay germline diagnosis. Risk of false negatives if somatic reversion and loss of heterozygosity is present. Inappropriate for patients post HSCT.	No

*Possible exclusions for which nail clippings should not be used includes patients post HSCT, particularly in the setting of active or recent graft-vs-host disease, due to risk of contamination with donor DNA, and MPN patients with marrow fibrosis, for which somatic variants have been detected [65]

^†^May be used as a means to rule out presence of germline variants when a rapid turn-around time is required, however, any detected variants require confirmatory germline testing. HSCT, allogeneic hematopoietic stem cell transplant

## Conclusion

Providers must maintain a high index of suspicion for HMs, even when personal or family history is not suggestive. When germline genetic testing and/or ancillary testing, such as TLs, is pursued, testing methods and tissue source considerations are crucial to ensure proper diagnosis and personalized management. Given the high frequency of deleterious germline variants found in HM patients across the age spectrum as well as in related and unrelated HSC donors, there is increasing evidence to support universal genetic testing for all patients with HM as well as potential HSC donors.

## Data Availability

No datasets were generated or analysed during the current study.

## Key References

- Feurstein S, Trottier AM, Estrada-Merly N, Pozsgai M, McNeely K, Drazer MW, et al. Germ line predisposition variants occur in myelodysplastic syndrome patients of all ages. Blood 2022;140:2533–48. 10.1182/BLOOD.2022015790,.
  - This study showed that germline variants in MDS patients undergoing allogeneic hematopoietic stem cell transplant are common across all age groups.
- Subbiah V, Kurzrock R. Universal Germline and Tumor Genomic Testing Needed to Win the War Against Cancer: Genomics Is the Diagnosis. J Clin Oncol 2023;41:3100–3. 10.1200/JCO.22.02833,.
  - This study provides evidence for universal germline testing.
- PDQ Cancer Genetics Editorial Board. RUNX1-Familial Platelet Disorder (PDQ^®^): Health Professional Version. PDQ Cancer Inf Summ 2002.
  - This study summarizes patient care considerations for patients with germline RUNX1 variants.
- Cunningham L, Merguerian M, Calvo KR, Davis J, Deuitch NT, Dulau-Florea A, et al. Natural history study of patients with familial platelet disorder with associated myeloid malignancy. Blood 2023;142:2146–58. 10.1182/BLOOD.2023019746,.
  - This is the first natural history study for patients with germline RUNX1 variants, expanding knowledge of associated phenotypes.
- Hendricks RM, Kim J, Haley JS, Ramos ML, Mirshahi UL, Carey DJ, et al. Genome-first determination of the prevalence and penetrance of eight germline myeloid malignancy predisposition genes: a study of two population-based cohorts. Leukemia 2024;39. 10.1038/S41375-024-02436-Y,
  - This genome first study significantly increased knowledge of the prevalence, penetrance and implications of eight germline myeloid malignancy syndromes.
- Alder JK, Hanumanthu VS, Strong MA, DeZern AE, Stanley SE, Takemoto CM, et al. Diagnostic utility of telomere length testing in a hospital-based setting. Proc Natl Acad Sci U S A 2018;115:E2358–65. 10.1073/PNAS.1720427115/SUPPL_FILE/PNAS.201720427SI.PDF.
  - This study found age-adjusted telomere lengths vary significantly with telomere biology disorder genotype/phenotype as well as age at presentation.
- Niewisch MR, Beier F, Savage SA. Clinical manifestations of telomere biology disorders in adults. Hematol Am Soc Hematol Educ Progr 2023;1:563–72. 10.1182/HEMATOLOGY.2023000490.
  - This article provides a comprehensive review of telomere biology disorders and practical guidelines for their management.
- Makishima H, Saiki R, Nannya Y, Korotev S, Gurnari C, Takeda J, et al. Germ line DDX41 mutations define a unique subtype of myeloid neoplasms. Blood 2023;141:534–49. 10.1182/BLOOD.2022018221,.
  - This very large international population-based study of myeloid neoplasms yielded new insights into DDX41-related myeloid malignancies and their unique characteristics in comparison to *de novo* cases.
